# ^1^H-NMR Spectroscopy Revealed *Mycobacterium tuberculosis* Caused Abnormal Serum Metabolic Profile of Cattle

**DOI:** 10.1371/journal.pone.0074507

**Published:** 2013-09-30

**Authors:** Yingyu Chen, Junfang Wu, Lingling Tu, Xuekai Xiong, Xidan Hu, Jiong Huang, Zhiguang Xu, Xiansong Zhang, Changmin Hu, Xueying Hu, Aizhen Guo, Yulan Wang, Huanchun Chen

**Affiliations:** 1 The National Key Laboratory of Agricultural Microbiology, Huazhong Agricultural University, Wuhan, Hubei, China; 2 College of Animal Science, Huazhong Agricultural University, Wuhan, Hubei, China; 3 College of Veterinary Medicine, Huazhong Agricultural University, Wuhan, Hubei, China; 4 Key Laboratory of Magnetic Resonance in Biological Systems, State Key Laboratory of Magnetic Resonance and Atomic and Molecular Physics, Wuhan Centre for Magnetic Resonance, Wuhan Institute of Physics and Mathematics, Chinese Academy of Sciences, Wuhan, Hubei, China; 5 Wuhan Keqian Animal Biological Products Co. Ltd., Wuhan, Hubei, China; 6 Xinjiang Academy of Animal Husbandry and Veterinary Institute, Urumqi, Xinjiang, China; University of Delhi, India

## Abstract

To re-evaluate virulence of *Mycobacterium tuberculosis* (*M. tb*) in cattle, we experimentally infected calves with *M. tb* and*Mycobacterium bovis*via intratracheal injection at a dose of 2.0×10^7^ CFU and observed the animals for 33 weeks. The intradermal tuberculin test and IFN-γin vitro release assay showed that both *M. tb* and *M. bovis* induced similar responses. Immunohistochemical staining of pulmonary lymph nodes indicated that the antigen MPB83 of both *M. tb* and *M. bovis* were similarly distributed in the tissue samples. Histological examinations showed all of the infected groups exhibited neutrophil infiltration to similar extents. Although the infected cattle did not develop granulomatous inflammation, the metabolic profiles changed significantly, which were characterized by a change in energy production pathways and increased concentrations of *N*-acetyl glycoproteins. Glycolysis was induced in the infected cattle by decreased glucose and increased lactate content, and enhanced fatty acid β-oxidation was induced by decreased TG content, and decreased gluconeogenesis indicated by the decreased concentration of glucogenic and ketogenic amino acids promoted utilization of substances other than glucose as energy sources. In addition, an increase in acute phase reactive serum glycoproteins, together with neutrophil infiltration and increased of IL-1β production indicated an early inflammatory response before granuloma formation. In conclusion, this study indicated that both *M. tb* and *M.bovis* were virulent to cattle. Therefore, it is likely that cattle with *M. tb* infections would be critical to tuberculosis transmission from cattle to humans. Nuclear magnetic resonance was demonstrated to be an efficient method to systematically evaluate *M. tb* and *M. bovi s*infection in cattle.

## Introduction

Tuberculosis (TB) is one of the most contagious human diseases. In 2011, there were an estimated 8.7 million new TB cases globally, resulting in approximately 1.4 million deaths [1]. The top 22 countries with high TB-burden account for approximately 81.6% of all new TB cases arising each year [2]. Most of these high TB-burden countries are developing countries [2], which have also experienced the great economical losses due to bovine TB (BTB) in cattle caused by a reduction in animal productivity as well as losses in domestic and international trade in addition to the cost of control and eradication programs [3]. Above all, TB is an important zoonotic infectious disease and BTB in cattle may be an important source of human TB. Although the proportion of human TB cases due to *M. bovis* in most developing countries remains largely unknown, it was speculated that the TB *bacillus* could account for as many as 10–15% of new human TB cases occurring in those countries [4] depending on the regions, patterns of cattle farming and trade, implementation of BTB control or eradication programs, and regional economic status, etc. [5], [6].

TB is caused by members of the *Mycobacterium tuberculosis* complex (MTBC), of which *Mycobacterium tuberculosis* (*M. tb*) and *Mycobacterium bovis* (*M. bovis*) are the most common pathogens. *M. bovis* resulted from genomic evolutionary reduction of *M. tb*, although the two species share 99.95% genomic identity [7]. *M. bovis* is the main pathogen of BTB and can cause TB in a wide range of animal hosts as well as humans. It is well recognized that *M. bovis* can infect humans through the respiratory route or the gastrointestinal tract via consumption of unpasteurized milk [5], [8], [9], [10]. *M. tb* is the main causative agent of human TB, but it is believed to cause only transient infections and appears to be avirulent in cattle [11], [12]. This early conclusion that *M. tb* is avirulent in cattle was based on the clinical observations and gross pathology of organs from cattle with 6–8 months of experimental infection [13].

The recent increase in the incidence of *M. tb*-infected cattle caused many public health officials to re-consider the food safety risk of *M. tb*-infected beef products. For example, approximately 27% of isolates from grazing cattle in central Ethiopia were *M. tb*-positive [14]. Furthermore, the investigators isolated *M. tb* strains from tissue lesions of cattle [6], [12], and the genotypes of these *M. tb* strains suggested TB transmission from humans to cattle [15]. These findings led Whelan et al. [11] to re-investigate *M. tb* virulence in cattle using two strains with known genomic sequences, *M. tb* H37Rv and *M. bovis* AF2122/97 [11]. Compared to earlier work [13], these investigators showed that both strains were equally infective and triggered strong cell-mediated immune responses in cattle as determined by interferon-gamma (IFN-γ) and a tuberculin skin test (TST), and that *M. tb* H37Rv could persist in infected cattle. Nevertheless, gross pathology examinations determined that *M. tb* H37Rv was avirulent to cattle [11]. Since *M. tb* is a very important human pathogen, it is necessary to extensively re-evaluate *M. tb* virulencein cattle using more sensitive methods.

In addition to traditional measurements of disease severity that are mainly based on pathology and immune response, nuclear magnetic resonance (NMR)/mass spectrometry (MS) profiling techniques combined with multivariate data analysis has recently been demonstrated as a powerful analytical tool for systematic evaluations of disease progression manifesting in metabolic changes, such as bacteremia [16] and schistosomiasis [17] in murine [18] and guinea pig models of TB [19]. Therefore, the aim of current investigation was to employ such powerful tools to re-evaluate *M. tb* virulence in cattle by monitoring metabolic responses in cattle to *M. tb* infection.

In this study, we artificially infected cattle for nearly 8 months with one *M. bovis* strain and two *M. tb* strains, including H37Rv and the clinical strain 1458, isolated from bovine lung tubercles. The evidence from immune responses, histopathological evaluations, and NMR-based serum metabolic profiles demonstrated that *M. tb* caused abnormal responses in cattle, which were similar to those caused by *M. bovis*. To the best of our knowledge, this is the first report to demonstrate both *M. tb* and *M. bovis* infections similarly induced abnormal metabolic responses in cattle. The results obtained in the current study undermined the traditional concept that *M. tb* is avirulent to cattle. More importantly, since *M. tb* is a much more important pathogen to humans than *M. bovis*, the evidence provided here suggests a need tore-evaluate the implementation of BTB control programs.

## Materials and Methods

### Cattle and grouping

Forty calves of local breed yellow cattle at approximately 6 months old were purchased from a local market in Urumqi, the capital of Xinjiang Uygur Autonomous Region in northwest China. The TB-free status was determined using a comparative TST according to the Manual of Diagnostic Tests and Vaccines for Terrestrial Animals published by the World Organization for Animal Health (OIE) [20] and levels of IFN-γ released by peripheral blood cells after tuberculin stimulation according to the product's instruction (BOVIGAM®; Prionics AG, Risch, Switzerland). The calves were encoded and labeled with ear tags and randomly divided into four groups (n = 10 each), including three infection groups and one mock-infected control group. Each group was housed in a separate room. The cattle were housed and slaughtered at the biosafety facility of Xinjiang Tecon Animal Husbandry Bio-Technology Co. Ltd. (Urumchi, China). The animal protocols were performed in strict accordance with the recommendations of Regulations for the Administration of Affairs Concerning Experimental Animals (1988) and the Regulations for the Administration of Affairs Concerning Experimental Animals (2005; Hubei, China). The study protocol was approved by the Committee on the Ethics of Animal Experiments of Department of Science & Technology of Hubei Province (Permit Number: SYXK (ER) 2010–0029).

### Bacterial Strains

The *M. tb* reference strain H37Rv (ATCC 27294) and *M. bovis* (ATCC 19210) were kindly provided by Dr. Chuanyou Li, Beijing Tuberculosis & Thoracic Tumor Research Institute (Beijing, China). The *M. tb* 1458 strain was clinically isolated from TB-positive cattle and stored in our laboratory according to previously described methods [21].

All strains were cultured to mid-log phase in Middlebrook 7H9 medium (Becton Dickinson and Company, Franklin Lakes, NJ, USA) supplemented with 10% oleic acid, albumin, dextrose, and catalase medium (OADC) (Becton Dickinson and Company) and 0.05% Tween 80 (Amresco Inc., Solon, OH, USA) with agitation in a biosafety level 3 facility at Huazhong Agricultural University (Wuhan, China) and stored at −80°C. The bacteria concentration (colony forming units (CFU)/mL) was determined using a plate counting assay as described previously [22].

### Experimental infection of cattle with *M. tb* and *M. bovis*

The infection experiment was performed between April 27, 2010 and January 12, 2011. Three groups of 10 calves each were infected with *M. tb* H37Rv, *M. tb* 1458, and *M. bovis*, respectively, at the dose of 2.0×10^7^ CFU in 2 mL via intratracheal injection. The negative control was mock-infected with an equal volume of phosphate-buffered saline (PBS). Blood samples were collected at regular 1-week intervals during the experimental period for monitoring of immune responses. All calves were sacrificed at 33 weeks post-inoculation (PI). The sera samples collected from each calf were frozen immediately and stored at –80°C for further NMR analysis and antibody detection. After a post-mortem examination, the pulmonary lymph nodes were collected and fixed for further histopathological examination, mycobacterium isolation, and immunohistochemical detection of the specific *M. tb* and *M. bovi s*antigen MPB83.

### Immune response detection

All calves were tested using *M. bovis* purified protein derivative (PPD-B) single intradermal skin test at 28 weeks PI. Skin tests were performed as described on the OIE website [20]. Briefly, cattle were injected with 2000 IU of PPD-B at the mid-neck and after 72 h, the skin-fold thickness was calculated and an increase of ≥4 mm was interpreted as a positive infection.

The heparinized blood was used for detection of in vitro IFN-γ release using a commercial kit (BOVIGAM®) according to the manufacturer's instructions (Prionics AG). Briefly, the whole blood samples were divided into three 1.5-mL aliquots, placed in separate wells, mixed with 100 μL of PPD-B, *Mycobacterium avium* PPD (PPD-A), or PBS, and incubated overnight in an atmosphere of 5% CO_2_. The supernatants in each well were harvested the next day for IFN-γ detection using a sandwich enzyme-linked immunosorbent assay (ELISA). A difference in optical density at 630 nm (OD_630_) between the PPD-B and PPD-A>0.1 was interpreted as positive.

Serum antibody levels were analyzed using an indirect ELISA kit (Wuhan Keqian Animal Biological Products Co., Ltd., Wuhan, China). Briefly, a fusion protein comprised of four *M. bovis* proteins (6-kDa early secreted antigenic target, culture filtrate protein-10, *Mycobacterium bovis* protein MPB-70, and MPB-83), which were used to coat the wells of 96-well ELISA plates. The serum samples were diluted to 1∶100 and then added to the wells. Goat immunoglobulin (Ig) G to cattle IgG Fc fragment coupled with horseradish peroxidase (HRP; Rockland Immunochemicals Inc., Gilbertsville, PA, USA) diluted to 1∶5000 was used as the secondary antibody. The OD_630_ values were measured and S/P values were calculated according to the formula:
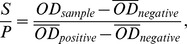
Where 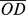 represents the average optical density of three repeats. The results were interpreted as positive when the S/P value was ≥0.17.

Interleukin (IL)-1β levels were detected in serum samples obtained at 7, 15, and 27 weeks PIusing a radioimmunoassay kit (North Biotechnology Institute, Beijing, China) at the North Biotechnology Institute (Beijing, China). Plasma samples obtained at 27 weeks PI were stimulated overnight with PBS and PPD-B, and then tumor necrosis factor-alpha (TNF-α) was detected using a homemade radioimmunoassay kit in the same institute.

### Post-mortem examination

At the end of the experiment, all calves were sacrificed and gross post-mortem examinations were conducted to detect lesions. A 35-point scoring system was used to grade gross pathological changes of the organs, tonsils, mandibular and pulmonary lymph nodes [23], and lungs, in which each lobule was scored 5 points as previously described [11].

The lung and pulmonary lymph nodes were collected and fixed immediately with 10% neutral phosphate-buffered saline (PBS) (pH 7.4) and formalin for histopathological and immunohistochemical examinations. Tissues were embedded in paraffin and cut into 4-μm-thick sections that were stained with hematoxylin and eosin stain for histopathological observations. The neutrophils were quantitatively analyzed using 10 fields of one randomly selected slide for each calf under light microscopy. The total neutrophil count for each group was compared.

### Bacterial detection and isolation

The lung, pulmonary lymph nodes, and tonsils were collected and treated with 4% NaOH, and mycobacteria were isolated using Lowenstein – Jensen medium and the further genotyping of the isolates were performed in a biosafety level 3 facility as described previously [6].

### Antigen detection

Immunohistochemical staining of the bacterial antigen MPB-83 in lymph node tissue was performed using in-house mouse monoclonal antibody to recombinant MPB-83 (mAb-MPB83) according to a previously described procedure [23]. Before detection, the specificity was confirmed by western blot analysis using *Mycobacteriumgraminis*, *Mycobacterium avium, Mycobacterium intracellulare*, *Mycobacteriumsmegmatis*, Madin-Darby bovine kidney (MDBK) cells, *Mycobacterium* subspecies paratuberculosis, *Escherichiacoli* from bovine origin, and Salmonella from bovine origin as the unrelated antigen control. The western blot procedure was performed as described previously unless otherwise specifically stated [22]. The bacteria were grown using conventional methods and the bacterial number was adjusted to approximately 10^8^ CFU/mL, while the MDBK concentration was adjusted to 2×10^6^/mL. The samples were separated by sodium dodecyl sulfate polyacrylamide gel electrophoresis, then the proteins were transferred to anitrocellulose membrane, which were blocked with 5% non-fat milk powder for 1h, and then MPB83 monoclonal antibody (dilution, 1∶2000) was added and the membrane was incubated overnight at 4°C. Next, HRP-labeled goat antibody against mouse IgG (Southern Biotech, Birmingham, AL, USA) was added and the membranes were incubated for 1 h. The nitrocellulose membrane was washed with tris-buffered saline and Tween 20 after each step. Finally, 1–2 mL of electro chemiluminescence detection solution A and B was slowly added and the membranes were incubated for 2 min at room temperature. A Kodak Image Station was used to detect chemiluminescent signals. The band image was converted to grayscale and analyzed with ImageJ software (imagej.en.softonic.com).

After staining, one slide for each calf was randomly selected and five fields for each slide were evaluated by light microscopy. A positive brown signal was quantitatively expressed as integrated optical density (IOD) and the average IOD per field for each calf was calculated using Image-Pro Plus 6.0 (IPP6) software (Media Cybernetics Inc., Rockville, MD, USA). The total IODs between each two groups were compared.

### Multiplex and real-time polymerase chain reaction (PCR)

The lung and pulmonary lymph node tissues were collected for both multiplex and real-time PCR analysis. Multiplex PCR was performed as previous described [6]. Seven pairs of primers specific to 16S rRNA, Rv0577, IS1561 (Rv3349c), Rv1510 (RD4), Rv1970 (RD7), Rv3877/8 (RD1), and Rv3120 (RD12) in the H37Rv genome (GenBank accession no.: NC_000962) were synthesized by Shanghai Sangon Biological Technology and Services Co. Ltd., (Shanghai, China). The amplification of PCR yielded products to all the seven primer sets indicated the presence of *M. tb*, whereas the amplification of 16S rRNA, RV0577, IS1561, and Rv3877/8 indicated the presence of *M. bovis*. Real-time PCR was performed according to the Chinese national standard GB/T 27639–2011. The sample tissues were completely homogenized and DNA was extracted. Each 25-μL reaction volume included 10mMTris-HCl (pH 8.3), 50 mMKCl, 2.5 mM MgCl_2_, 2 mM dNTP, 200 nM of each primer. 0.1nM probe, 5% dimethyl sulfoxide, 5% glycerin, 0.4 Uuracil DNA glycosylase, 1U *Taq* 6Polymerase, and 5 µL DNA. Reactions were performed in an ABI 7500 Real-Time PCR System (Applied Biosystems, Foster City, CA, USA) under the following conditions: 50°C for 2 min, 95°C for 4 min, followed by 40 cycles of 95°C for 10 s and60°C for 45 s. The fluorescent signals were collected at 60°C during the elongation step.

### Sample preparation for metabonomics by^1^Hnuclear magnetic resonance (NMR) spectroscopy

The TB-infected and control calves were sacrificed and blood samples were collected and then centrifuged. A 200-μL aliquot of the serum fraction was mixed with 400 μL of saline solution containing 50% D_2_O, and 30 mM phosphate buffer (pH 7.4). All mixed solutions were vortexed and centrifuged and a total of 500 μL of the supernatant was transferred to 5-mm NMR tubes for NMR acquisition.

The ^1^H NMR spectra from serum samples were acquired at 298K using a Bruker AVIII 600-MHz NMR spectrometer (BrukerBioSpin GmbH, Rheinstetten, Germany) equipped with a cryogenic probe and operated at a proton frequency of 600.13 MHz. Standard water-suppressed one-dimensional NMR was performed and recorded using sequence [recycle delay (RD)-90°-*t_1_*-90°-*t_m_*-90°-ACQ] setting t1 to 3 μs and a mixing time (*t_m_*) of 100 ms. Water suppression was achieved with irradiation of the water peak during the RD (2s) and mixing time (*t_m_*). The 90° pulse length was adjusted to ∼10 μs. A total of 64 transients were collected into 32 k data points for each spectrum with a spectral width of 20 ppm. Furthermore, two additional pulse sequences, namely Carr–Purcell–Meiboom–Gill [24] (CPMG) and diffusion edited spectroscopy [25] were also applied to focus on low and high molecular weight components of the serum profile, respectively. The spin-spin relaxation delay (2nτ) of 70 ms was used for serum CPMG spectra acquisition.

For signal assignment purposes, selected samples were conducted with a series of standard two-dimensional (2D) spectra, including ^1^H-^1^H correlation spectroscopy (COSY), ^1^H-^1^H total correlation spectroscopy (TOCSY), ^1^H-^13^C heteronuclear single quantum correlation (HSQC), and ^1^H-^13^C heteronuclear multiple bond correlation spectra (HMBC). Assignments of the spectra peaks were made based on the literature [26], [27] with the aid of statistical total correlation spectroscopy (STOCSY) and confirmed with the ranges of 2D NMR spectra.

### Statistical Analysis

For the NMR spectra, all free induction decays were multiplied by an exponential function equivalent to a 1-Hz line-broadening factor prior to Fourier transformation. After manual corrections for phase and baseline distortions, the spectra were calibrated to an anomeric proton signal from α-glucose (δ5.23) for sera. The processed NMR data were reduced into spectral regions of 0.002 ppm in the environment of AMIX package (BrukerBioSpin GmbH). Regions containing water resonances (δ4.43-5.18) were excluded. The resonances at δ3.65 and δ1.18 arising from ethanol used for sterilization were also eliminated.

All reduced data matrices were imported into SIMCA-P software (Umetrics AB, Umea, Sweden) for Principle Component Analysis (PCA) [28], Partial-Least-Square Discriminant Analysis (PLS-DA) [29], and Orthogonal PLS-DA (O-PLS-DA) [30]. Of these, PCA was used to identify general trends and outliers. PLS-DA was used to maximize the difference between the compared groups (scaled to unit variance) [31] and O-PLS-DA was used to further extract useful information relevant to *M. tuberculosis* infection to identify significant metabolites that contributed to group separation. All models were cross validated using a 7-fold method and further evaluated using permutation tests (200 permutation numbers) [32], [33]. Interpretation of the model was facilitated by back-scaled transformation of the loadings with incorporated color-coded correlation coefficients of the metabolites responsible for the differentiation using in-house-developed MATLAB scripts. A correlation coefficient higher than the cut-off value indicated that the metabolite significantly contributed to group separation.

Immune response data (such as IFN-γ and antibody levels) were analyzed using SPSS statistical software (SPSS Inc., Chicago, IL, USA). Values are presented expressed as means ± the standard error of the mean (SEM). Multiple comparisons were made using one-way analysis of variance (ANOVA). A probability (*p*) value <0.05 was considered statistically significant (*) and a *p*-value <0.01 was considered very significantly (**).

## Results

### The positive conversion rate of cattle after inoculation of *M. tb* and *M. bovis* as determined by several methods

The PPD skin test was performed at 16 weeks PI. All 10 calves in the control group were PPD-negative. The proportions of positive calves in the *M. tb*H37Rv, *M. tb*1458, and *M. bovis* groups were 10/10, 4/10, and 4/10, respectively. The infection rate was higher in the *M. tb*H37Rv group compared to the *M. tb*1458 and *M. bovis* groups.

**Figure 1:**
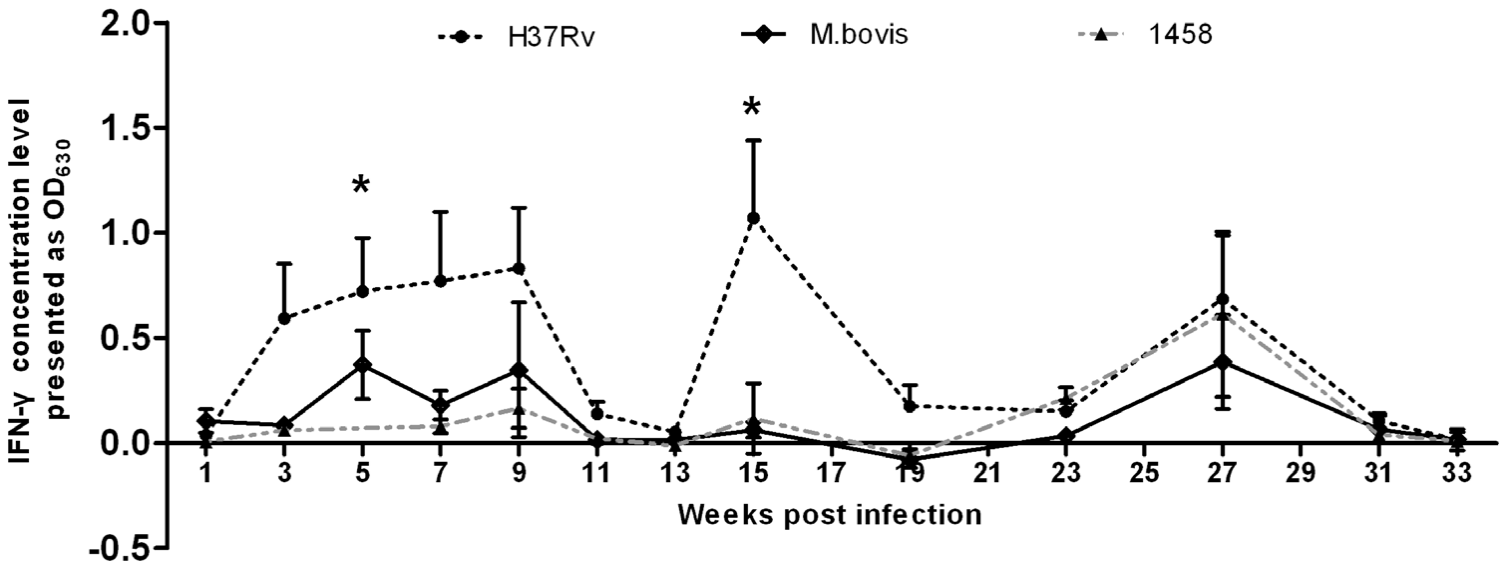
Experimental infection of calves with *M. tb*H37Rv, *M.tb*1458 of bovine origin and *M. bovis* induced IFN-γ production after in vitro stimulation with bovine PPD. The IFN-γ concentrations, which were measured by BOVIGAM® ELISA kit and expressed as OD_630_ values, fluctuated with the time course of infection. The OD_630_ values at each timepoint were presented as mean ± SEM. On week 7PI, there were significant differences between the *M. tb*H37Rv- and *M. tb*1458-infected groups, while at week 15 PI, the difference were statistically significant between the *M. tb*H37Rv vs.1458, and *M. tb*H37Rv vs. *M. bovis* infected groups.

IFN-γ levels in peripheral blood fluctuated throughout the experimental period, although IFN-γ response increased gradually at the beginning of the infection and climbed to peak levels at about week 10 PI, and then decreased gradually (Figure 1). Specifically, a few calves in the *M. tb*H37Rv (1/10) and *M. bovis* (3/10) groups, but none of calves (0/10) in the *M. tb*1458 group, showed a positive response to IFN-γ at week 1 PI (Table 1). However, at week 10 PI, most of the infected calves were PPD-positive with ratios of positive calves in the *M. tb*H37Rv, *M. tb*1458, and *M. bovis* groups of 10/10, 7/10, and 9/10 respectively, which were higher than those of PPD-positive calves detected at week 16 PI. In addition, *M. tb*H37Rv infection induced the strongest response among the three infected groups (Figure 1). When the results of the PPD skin test and IFN-γ in vitro release assay were combined, all calves administered a mycobacterium inoculation became positive, while the mock-infected calves maintained a negative status.

**Table 1:** Time and proportions of calves that turned positive to IFN-γ assay for bovine TB.

	Weeks post inoculation									
Groups	1	2	7	9	10	11	12	13	15	16
*M.tb*H37Rv	1/10	6/10	8/10	9/10	10/10	10/10	10/10	10/10	10/10	10/10
*M.tb*1458	0/10	2/10	5/10	6/10	7/10	7/10	6/10	7/10	6/10	6/10
*M. bovis*	3/10	4/10	7/10	7/10	9/10	9/10	9/10	9/10	9/10	9/10
Average	13%	40%	67%	73%	87%	87%	83%	87%	83%	83%

**Figure 2:**
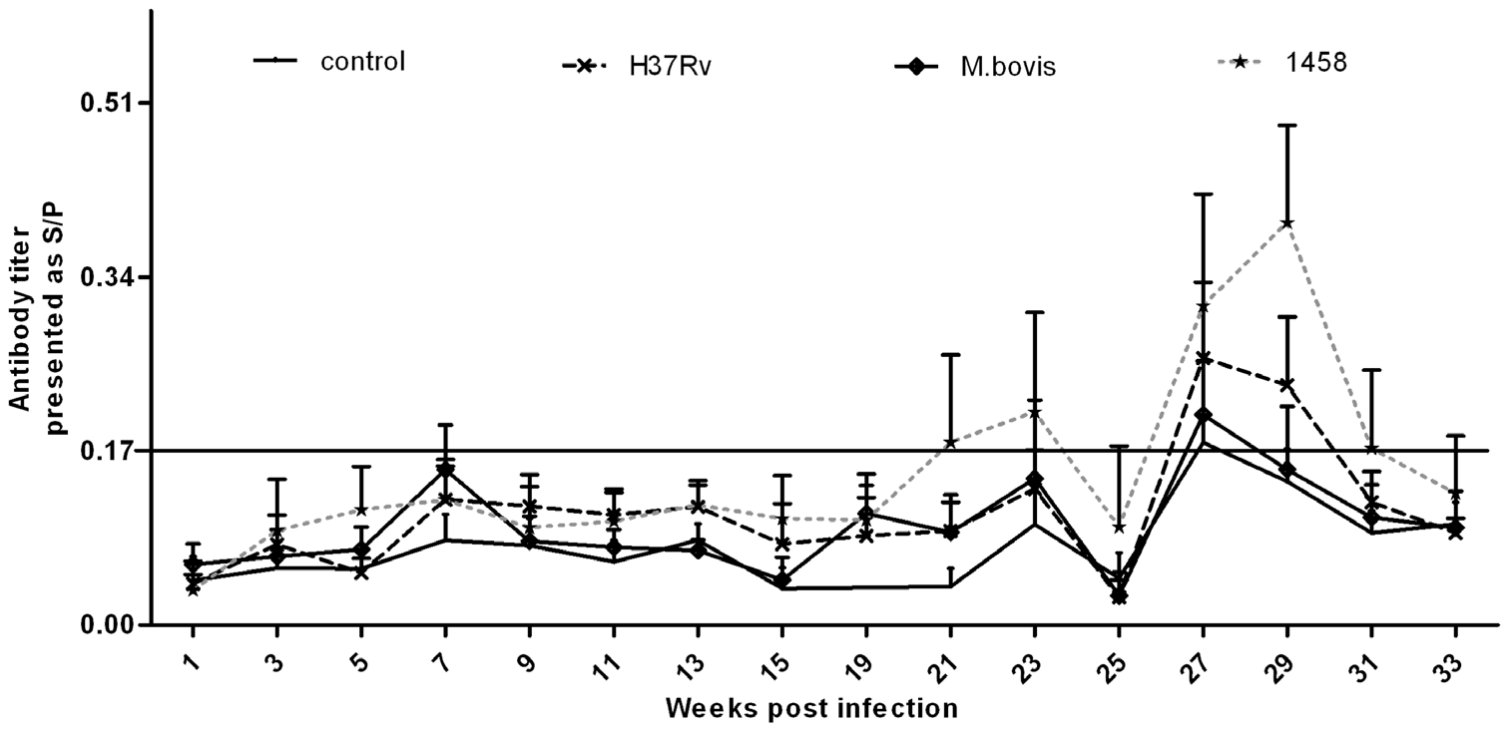
Experimental infection of calves with *M. tb*H37Rv, *M.tb*1458 of bovine origin, and *M. bovis* induced serum IgG production. The IgG concentrations, which were measured by indirect ELISA and expressed as OD_630_ values, apparently increased at the late stage of infection and were maintained for short time (weeks 28–29). The OD_630_ values at each timepoint were presented as mean ± SEM.

Furthermore, the apparent increase in IgG levels occurred at very late stage of infection, ranging from week 28 to 31 PI in all of the infected groups, although the IgG levels were less than the cut-off limits at the other time points (Figure 2).

### Bacterial detection

The mycobacteria were isolated from a mixed culture lung lymph node and tonsil tissues from two calves in the *M. tb*-infected groups, one in the *M. tb*1458 group, and one in the *M. tb*H37Rv group. Further characterization by multiplex PCR and spoligotyping [6] confirmed that these two isolates recovered from the *M. tb*1458 and H37Rv groups were members of the *M. tb* Beijing family and *M. tb* H37Rv strain, respectively.

**Figure 3:**
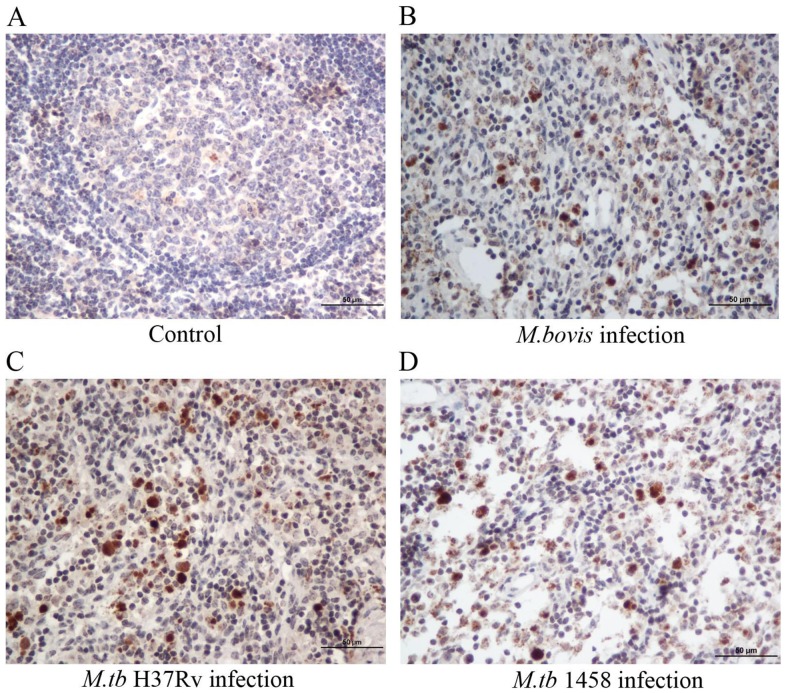
Histopathological observations of the tissues. An increase in neutrophil infiltration and lipofuscin-containing macrophages was observed in cattle lymph node tissue in groups infected with *M. tb*H37Rv, *M. tb*1458 of bovine origin, and *M. bovis*. The cells were stained with hematoxylin and eosin and observed via light microscopy. The black solid arrows indicate neutrophils and the double line arrow indicate lipofuscin-containing macrophages. Only B shows both cell types because it was difficult to find both co-existing in the same field.

**Figure 4:**
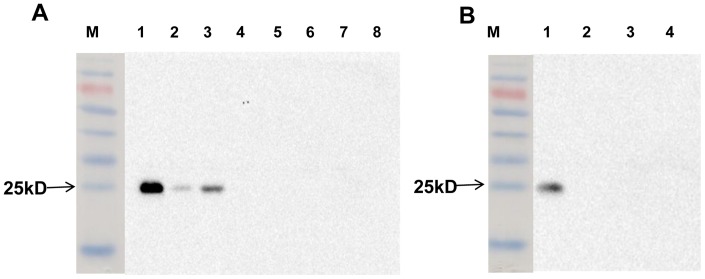
Western blot analysis to detect the specific MPB83 monoclonal antibody. (A) M, prestain marker; Lane 1, *M. bovis*; Lane 2, *M. tb*1458; Lane 3, H37Rv; Lane 4, *M.graminis*; Lane 5, *M. avium*; Lane 6, *M. intracellulare*; Lane7, *M. smegmatisc*; and Lane 8, MDBK cells. (B) M, prestain marker; Lane 1, H37Rv; Lane 2, *M.* paratuberculosis; Lane 3, *E.*coli from bovine origin; and Lane 4, Salmonella from bovine origin. The arrows indicate the MPB 83 protein.

In addition, immunohistochemical staining revealed the specific bacterial antigen MPB83 of *M. tb* and *M. bovis* in lymph node tissues sections (Figure 3). Western blot analysis showed that mAb MPB83 was very specific to *M. bovis* and *M. tb*, and only *M. bovis* and *M. tb* (including strains H37Rv and 1458) reacted to mAb MPB83, although the band of *M. bovis* was stronger than those of the *M. tb* strains on the blotted membrane, while all the other proteins, including *M.graminis*, *M. avium, M. intracellulare*, *M.smegmatis*, *M.paratuberculosis*, *Escherichiacoli* and Salmonella strains of bovine origin, and MDBK cells as tissue protein controls, did not react to mAbMPB83 (Figure 4), which indicated that MPB83 was specific to *M. tb* and *M. bovis*.

Immunohistological analysis of the stained tissue slides was performed and the total IODs of the brown cells of each group were counted automatically using IPP6 software based on the brown color intensity. The infected groups had significantly higher IOD values than the control group (*p*<0.01). However, there was no difference in IOD values among the three infected groups (*p*>0.05) (Table 2). Our results confirmed that both *M. tb* (H37Rv and 1458) and *M. bovis* successfully infected the cattle and resided in tissues, including the lymph nodes, although the multiplex and real-time PCR results were not always in agreement.

**Table 2:** Comparison of integrated optical density (IOD) for MPB83 positive cells among groups.

Groups	IOD (mean ± SEM) per group	No. of fields observed
Control	43.8±34.2^A^	100
*M. tb*H37Rv	10877.6±8037.9^B^	100
*M. tb*1458	18459.7±9855.1^CB^	100
*M. bovis*	11400.6±3337.1^DBC^	100

Note: the same superscript letters in the second column represent no significant difference (*p*>0.05) between the two rows, whereas the different superscript lowercase letters represent significant differences between the two rows (*p*<0.05), while the superscript uppercase letters indicate very significant differences (*p*<0.01).

### Gross pathological and histopathological observation of infected calves

**Figure 5:**
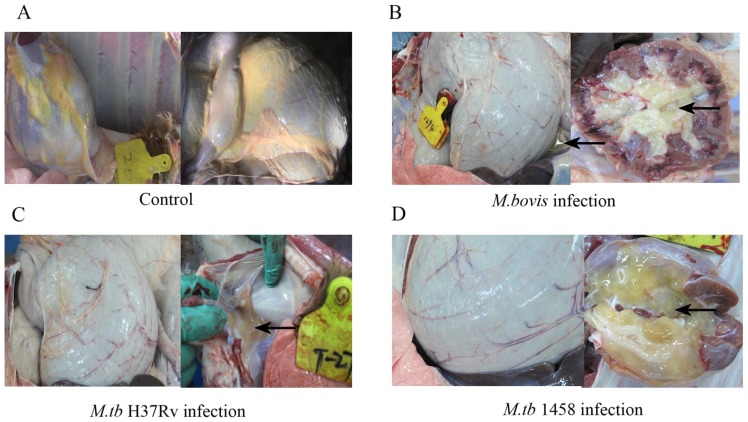
Gross pathological observations of the calves. The subcutaneous adipose tissues were very thick in the negative control group (A). The calves had thinner layers of subcutaneous adipose tissue and obviously gelatinous materials after infection with *M.bovis* (B), *M. tb*H37Rv (C), and *M. tb 1458*of bovine origin (D). Arrows indicate the gelatinous tissue material.

Not all of the infected calves exhibited typical TB lesions, such as tubercles, in tissues during the post-mortem gross examinations. The only apparent difference between the infected and negative control animals was the change in subcutaneous adipose tissue, as the infected calves had thinner layers of the subcutaneous adipose tissue than the control calves (Figure 5). Gelatinous materials other than adipose tissues were easily observed within some fat-rich areas of the stomach, kidneys, renal pelvis, and heart in the infected cattle, which is a sign indicative of adipose consumption. The evidence combined qualitatively suggested that the infected calves experienced a type of malnutrition.

**Figure 6:**
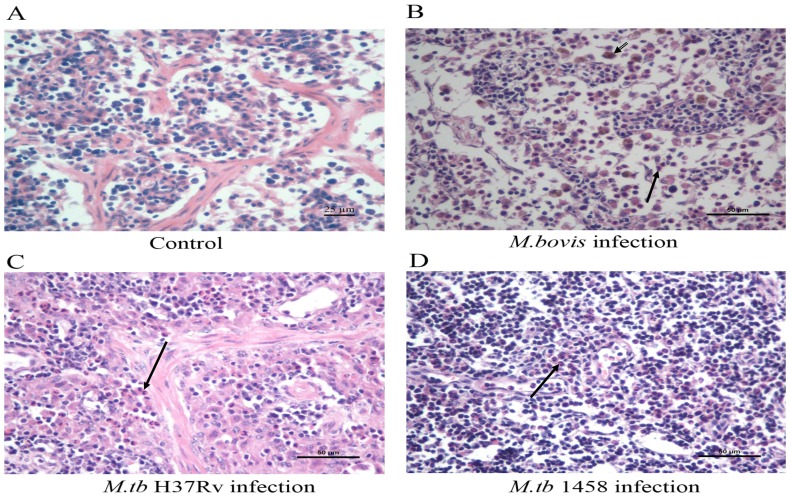
Immunohistochemical detection of a specific antigen against *M. tb* and *M. bovis* in lymph node tissues. The antigen MPB83 for *M. tb* and *M. bovis* in the lymph node tissues of calves was observed after immunohistochemical staining. There were no positive cells in the negative control group (A). The MPB83-positive cells were stained brown in the groups infected with *M. bovis* (B), *M. tb*H37Rv (C), and *M. tb 1458*of bovine origin (D).

In addition, there were no typical histopathological changes caused by BTB infection, such as tubercles or multinucleated giant cells in the tubercles in the lungs, livers, kidneys, lymph nodes, or tonsils. However, the foci of neutrophil infiltration and lipofuscin-containing macrophages were observed in various sites in the lymph nodes and tonsils of the infected animals (Figure 6B). The neutrophils within the lymph node tissues were further manually counted. Compared to the control group, all of the infected groups exhibited significantly increased neutrophil infiltration (*p*<0.01) (Figure 6 and Table 3). Notably, there were no differences in neutrophil counts between the cattle infected with *M. bovis* and with *M. tb*1458 (*p* = 0.07); nonetheless, the neutrophil counts in the groups of both strains were significantly higher than that in the *M. tb* H37Rv group (*p*<0.01).

**Table 3:** Comparison of neutrophil numbers among groups.

Groups	Cells (mean ± SEM)**per field	No. of fields observed
Control	5.3±0.6^A^	50
*M. tb*H37Rv	8.1±0.7^B^	50
*M. tb*1458	16.0±1.7^C^	50
*M. bovis*	12.4±1.0^DC^	50

Note: the same superscript letters in the second column represent no significant difference (*p*>0.05) between two rows, whereas the different superscript lowercase letters represent significant differences, (*p*<0.05), while the superscript uppercase letters indicate very significant differences (*p*<0.01).

### Concentrations of proinflammatory cytokines IL-1β and TNF-α

**Figure 7:**
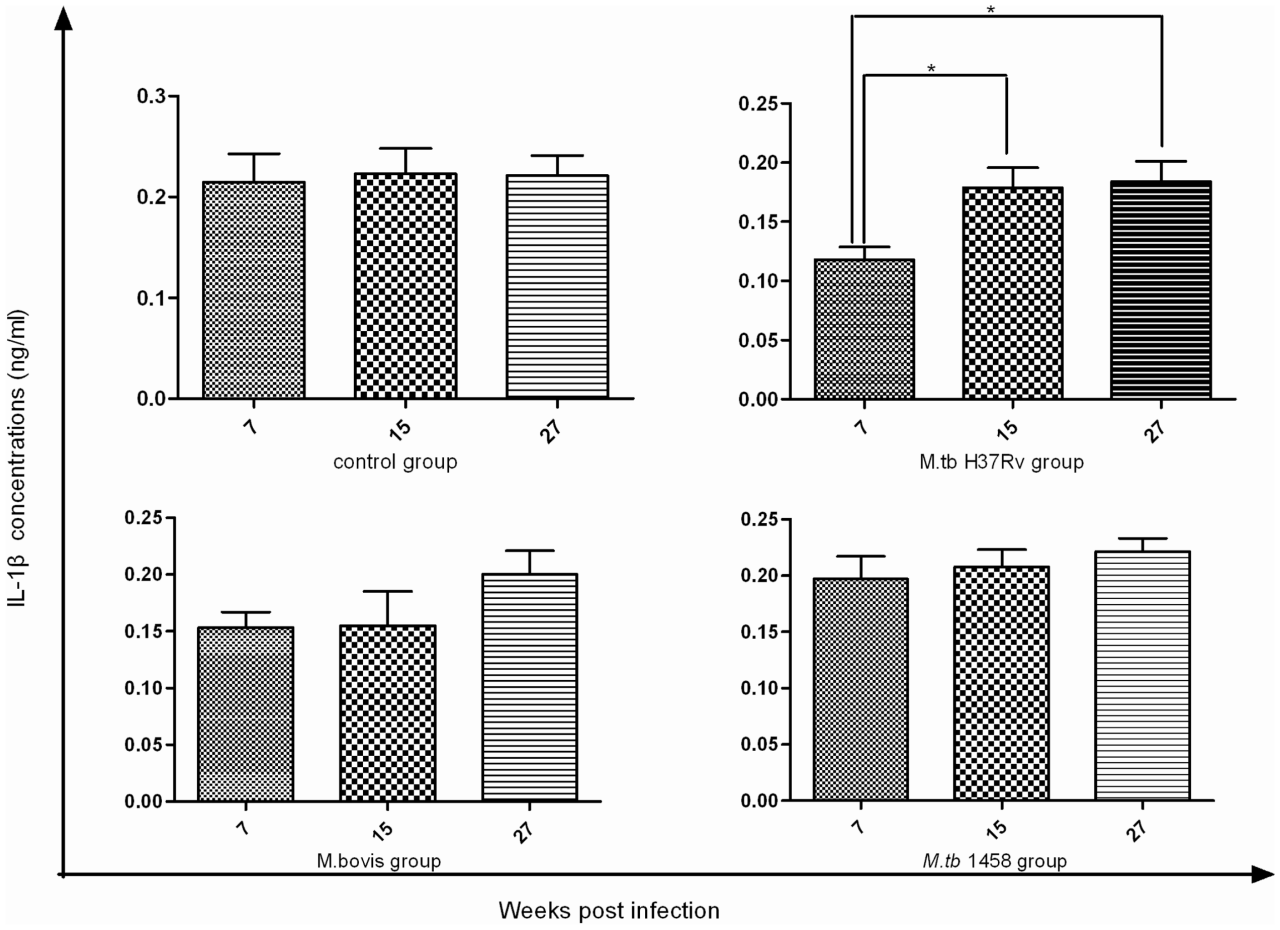
Serum IL-1β detection in all groups. IL-1β concentrations were detected at weeks 7, 15, and 27 PI. in the H37Rv group, the serum IL-1β concentration was enhanced and there was significant difference between week 7 vs. 15 and week 7 vs. 27, while there was no significant increase in the other groups during the same periods.

IL-1β concentrations in the infected groups showed continuous increases from week 7 to week 27 PI. In contrast, IL-1β levels in the control group remained unchanged during this period. The differences between weeks 7 and 15 PI and weeks 7 and 27 PI were statistically significant in the *M. tb*H37Rv group (*p*<0.05, *p*<0.05, respectively), while there were no significant difference in the other two infected groups during this period (Figure 7). TNF-α concentrations were not detected in anysamples.

### Metabolic profiles of bovine serum profiles

**Figure 8:**
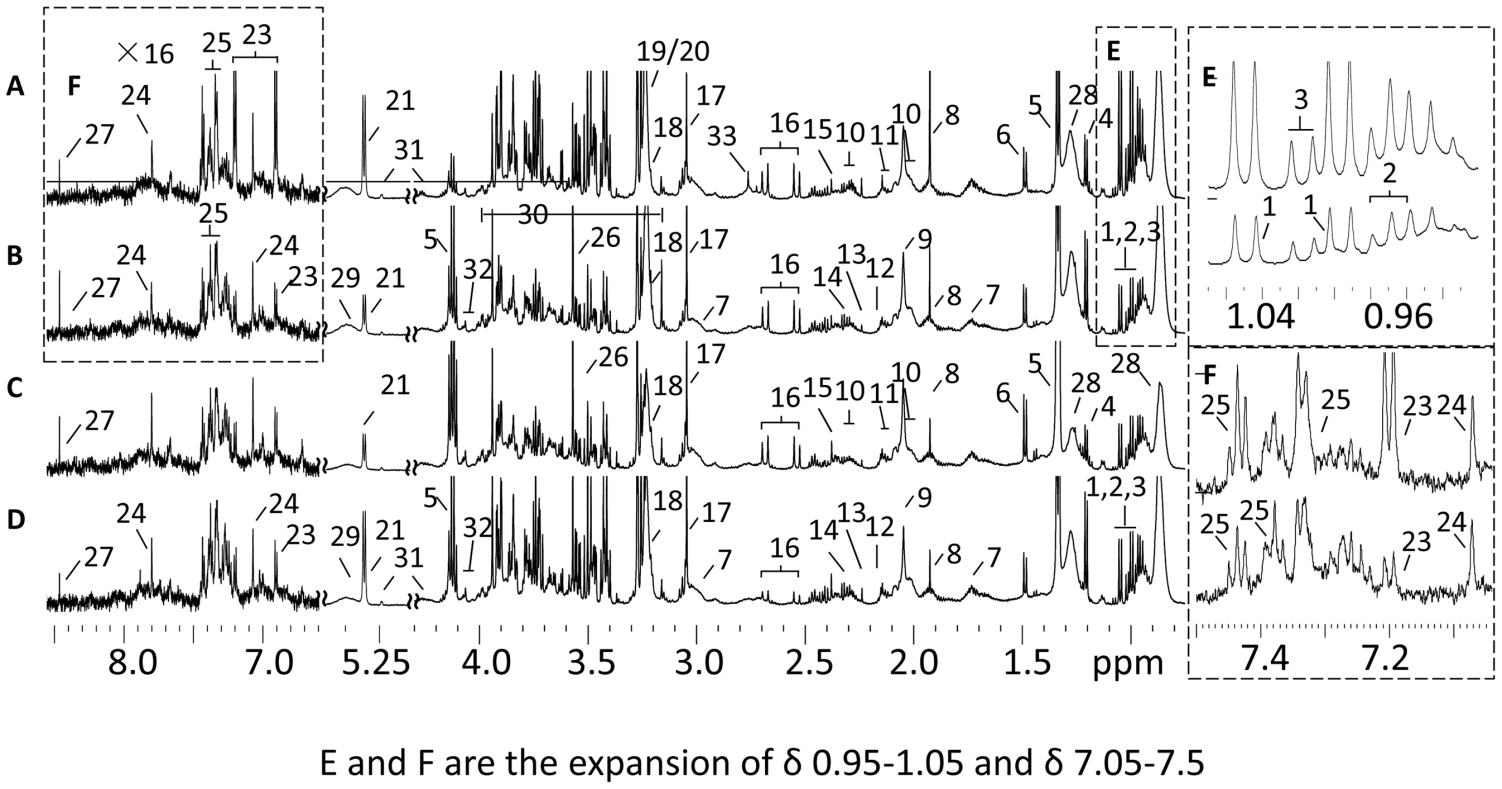
Typical 600-MHz ^1^H NMR CPMG spectra of plasma. A–D, represent controls (A) and calves infected with *M. bovis* (B), H37Rv (C), and *M. tb*1458 (D), respectively. The spectral regions of δ 6.5–8.5 were expanded 16 times. Keys: 1, valine; 2, leucine; 3, isoleucine; 4, 3-hydroxybutyrate; 5, lactate; 6, alanine; 7, lysine; 8, acetate; 9, N-acetyl-glycoprotein; 10, glutamate; 11, glutamine; 12, methionine; 13, acetone; 14, acetoacetate; 15, pyruvate; 16, citrate; 17, creatine; 18, choline; 19, phosphorylcholine; 20, glycerylphosphorylcholine; 21, glucose; 22, fumarate; 23, tyrosine; 24, 1-methyl-histidine; 25, phenylalanine; 26, glycine; 27, formate; 28, lipid; 29, unsaturated fatty acid; 30, glucose and amino acids a-CH resonances; 31. Triacylglycerol; 32, myo-inositol; and 33, unknown.

Typical ^1^H CPMG NMR profiles of the sera obtained from the infected and control groups on the last day of the experiment were analyzed (Figure 8). More than 30 metabolites were identified based on the literature data and confirmed with a series of 2D NMR experiments (COSY, TOCSY, HMBC, and HSQC). The endogenous metabolites identified in serum spectra included glucose, lipids, ketone bodies (acetone, β-hydroxybutyrate, and acetoacetate) and a number of amino acids (valine, leucine, isoleucine, tyrosine, and phenylalanine). Visual inspection of the spectra revealed lower levels of glucose and branch chain amino acids (BCAAs) and higher levels of *N*-acetyl glycoprotein in the infected groups. Further extraction of metabolites associated with the infection was performed on the NMR data using multivariate data analysis.

### Metabolic variations between the control and different MTBC strains infection

**Figure 9:**
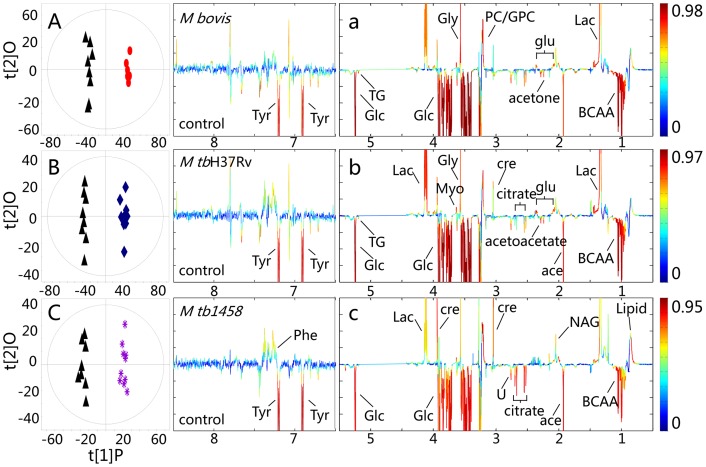
OPLSDA comparison of plasma spectra between the control and infected groups (*M. bovis*, *M. tb*H37Rv, and *M. tb*1458). In panels A, B, and C, the triangle (▴) represents the control, the black circle (•) represents *M. bovis*infected group, the diamond (◊) represents the *M. tb*H37Rv-infected group, the asteric (*) represents the *M. tb*1458-infected group; In panels a, b, and c, the upward and downward peaks, respectively, denote the elevated and decreased metabolites in the infected calves with hot colored metabolites contributing more significantly to the class discrimination than the cold colored metabolites.

PCA score plots were constructed initially for an overview of the dataset and to visualize the outliers of all samples. To further investigate the associations of metabolites or metabolic pathways with *M. tb* and *M. bovis* infections, O-PLS-DA models were constructed using NMR data as the X-matrix and group information as the Y-matrix. The O-PLS-DA score plots showed (Figure 9A–9C) clear separations between the *M. bovis*, *M. tb*H37Rv, and 1458 strain groups and the corresponding controls. The O-PLS-DA model was validated using a seven-fold cross-validation strategy, rigorous permutation tests, and CV-ANOVA tests. The color-coded co-efficient plots (Figure 9a–9c) generated using the O-PLS-DA model revealed detailed metabolic changes induced by bacterial infection, as indicated by the coefficients summarized in Table 4. The upward and downward peaks, respectively, denote elevated and depleted metabolites in the sera of infected calves with red colored metabolites contributing more significantly to the class discrimination than the blue colored metabolited. The serum samples showed significant depletion of acetone, acetoacetate, glucose, triglycerides (TGs), and a range of amino acids, such as BCAAs, tyrosine, methionine, together with elevation of lactate, creatine, and membrane-related metabolites, such as phosphorylcholine (PC), in the *M. bovis*-and *M. tb*H37Rv-infected groups. Compared to the controls, the metabolic changes in the sera samples obtained from the *M. tb*1458-infected calves showed no significant changes in acetone and acetoacetate concentrations (Table 4).

**Table 4:** Significant changes in serum metabolites from TB-infected cattle compared to non-infected controls.

		Fold changes		
Metabolites	Chemicalshift (ppm)	*M. bovis*	*M.tb H37 Rv*	*M.tb 1458*
Glucose	5.23	−4.06	−3.2	−1.99
Lactate	1.33	3.43	3.94	2.3
Acetoacetate	2.29	−1.24	−1.21	−1.1
TG	5.18	−1.99	−1.57	−1.28
Lipid	0.84	-	-	1.29
Creatine	3.93	-	1.17	1.38
Glycine	3.55	1.65	1.58	1.47
Isoleucine	1.01	−1.76	−1.61	−1.37
Leucine	0.96	−1.4	−1.32	−1.15
Methionine	2.15	-	-	−0.75
Tyrosine	7.20	−2.19	−2.02	−1.99
Valine	1.04	−2.24	−2.05	−1.63
Acetate	1.92	−2.2	−2.66	−3.04
Citrate	2.54	−1.5	−1.44	−2.14
Choline	3.20	1.46	1.52	1.82
PC/GPC	3.21	1.85	1.77	1.74
Acetone	2.23	−1.43	−1.38	-
Myoinositol	4.06	1.22	1.26	1.18
N-acetyl glycoproteins	2.04	1.25	1.21	1.21
Unknown	2.76	−1.72	−1.66	−1.66

Note: Data value represents significant fold changes in Tb-infected calves compared to controls. – indicates no changes in the level of significance (p>0.05).

## Discussion

Although *M. bovis* is the main pathogen of BTB, *M. tb* has also been increasingly found in TB-positive cattle [12], [14], [34]. Since *M. tb* was previously considered avirulent in cattle [11], the risk of *M. tb*-infected cattle to public health was neglected. These conclusions were solely based on the absence of observable clinical signs and typical gross pathology. However, the occurrence of clinical signs and typical gross pathology was affected by many factors in the experimentally infected calves. First, the gross pathology results were affected by breed of the calves. For instance, Friesian cattle were reportedly more sensitive to *M. bovis* and it was easier to develop gross pathological lesions than in a local breed in an experimental infection study conducted in Ethiopia [35]. Secondly, the infection routes, doses, and disease duration played important roles in disease progression [36]. Based on the above findings, we performed the current investigation with a view that the current parameters defining TB cases based on clinical signs and gross pathology might be insensitive and insufficient.

To improve TB detection sensitivity, NMR-based metabonomics were used for systematical evaluations of disease progression in the current study. This method was recently shown to be a powerful analytical tool to reveal abnormalities in mice and guinea pigs following *M. tb* infection [18]. Contrary to previous findings, we demonstrated that *M. tb* and *M. bovis* similarly caused subclinical abnormalities in cattle. This conclusion challenged the traditional concept that *M. tb* is avirulent to cattle and should convince governmental entities and commercial interests in the beef industry to take more effective measures to control BTB since humans are much more susceptible to *M. tb* than *M. bovis*.

### *M. tb* strains and *M. bovis* similarly infected cattle during the experimental infection

We infected the calves via intratracheal injection at a dose of 2.0×10^7^ CFU and observed the animals for 33 weeks. The strains *M. tb*H37Rv and *M. bovis*, but not *M. tb* Beijing family strain 1458, the dose, administration route, and observation time were previously reported in the literature to be effective in experimental infections of mice [37] and cattle [11]. However, experimental infections with the *M. tb* strains and *M. bovis* failed to induce typical gross tubercles in the organs and granulomas in tissue sections from cattle. However, these differences may have occurred due to breed differences. Here, we used local yellow cattle, although there was evidence that this breed was more resistant to the MTBC members than were foreign breeds, such as Friesian and Holstein dairy cattle, as reported previously by other investigators who discovered that cattle breeds influenced susceptibility to BTB in Ethiopia [35]. On the other hand, we cannot exclude the possibility that in vitro passage of *M. bovis* and *M. tb* strains attenuated the virulence of these strains.

Based on the above results, we further explored the subclinical evidence to determine the success of the infection at the cellular and molecular levels. The results of the immune response, the IFN-γ values, and antibody production concluded that these bacteria successfully infected the cattle. IFN-γ production was induced at the early stage of infection, while antibody was produced at a very late stage. These results were in agreement with previous findings showing that the antibody response to *M. tb* or *M. bovis* lagged behind cellular immunity after infection and was positively correlated to the infection course [38]. Surprisingly, *M. tb*H37Rv tended to stimulate a stronger response than other strains, for example, at week 15 PI, the *M. tb*H37Rv-infected group exhibited a significantly stronger IFN-γ response than the *M. bovis*-and *M. tb*-1458 infected groups. However, these mechanisms remain to be investigated.

A second typical response of the host after *M. tb* infection is granulomatous inflammation and the subsequent formation of granulomas, in which the immune system walls off the bacteria, but is unable to eliminate them. Murine TB models were used to show that during the early stages of infection, neutrophils and inflammatory monocytes were recruited to the infected sites and regulated the accumulation of Th1 cells in *M. tb*-infected lung tissues via chemokine production [39], [40]. Although we did not observe typical granuloma formation in the tissue sections, we did observe significant neutrophil infiltration, representative of an early stage of inflammation, and the increase in IL-1β content and infection duration are important mediators of the inflammatory response. Furthermore, there was no difference in neutrophil infiltration among the three infected groups, indicating that both *M. tb* and *M. bovis* successfully induced early inflammation responses. Because IL-1β and neutrophil infiltration significantly increased in this study, but yet no TNF-α or typical TB lesions were induced, we concluded that the infected animals only developed mild inflammatory responses.

Although we only recovered bacteria from a few infected individuals, the presence of specific antigens in target cells was detected. MPB83 is a specific gene that is highly expressed in virulent *M. bovis*. Although its homologue MPT83 in *M. tb* is expressed at lower levels when grown in vitro, this protein is also highly immunogenic during infection with live bacteria [41]. Hence in the current study, we used MPB83 monoclonal antibody to quantitatively assess the existence of antigens in lung and lymph node tissues by immunohistochemical methods. Our results showed that calves infected with three strains were similarly positive to the antigen detection, but no statistical difference was observed among the three infected groups. This finding demonstrated that both *M. tb* and *M. bovis* had similar abilities to invade cattle tissue.

Based on the above evidence collectively, we concluded that *M. tb*H37Rv, *M. tb* Beijing family strain 1458, and *M. bovis* could equally infect cattle, induce immune responses, and maintain infections in cattle for 33 weeks. These findings are also supported by the metabolic alterations of sera obtained from the infected calves as discussed in the following section.

### Energy deprivation supported clinical malnutrition or wasting of the infected cattle

**Figure 10:**
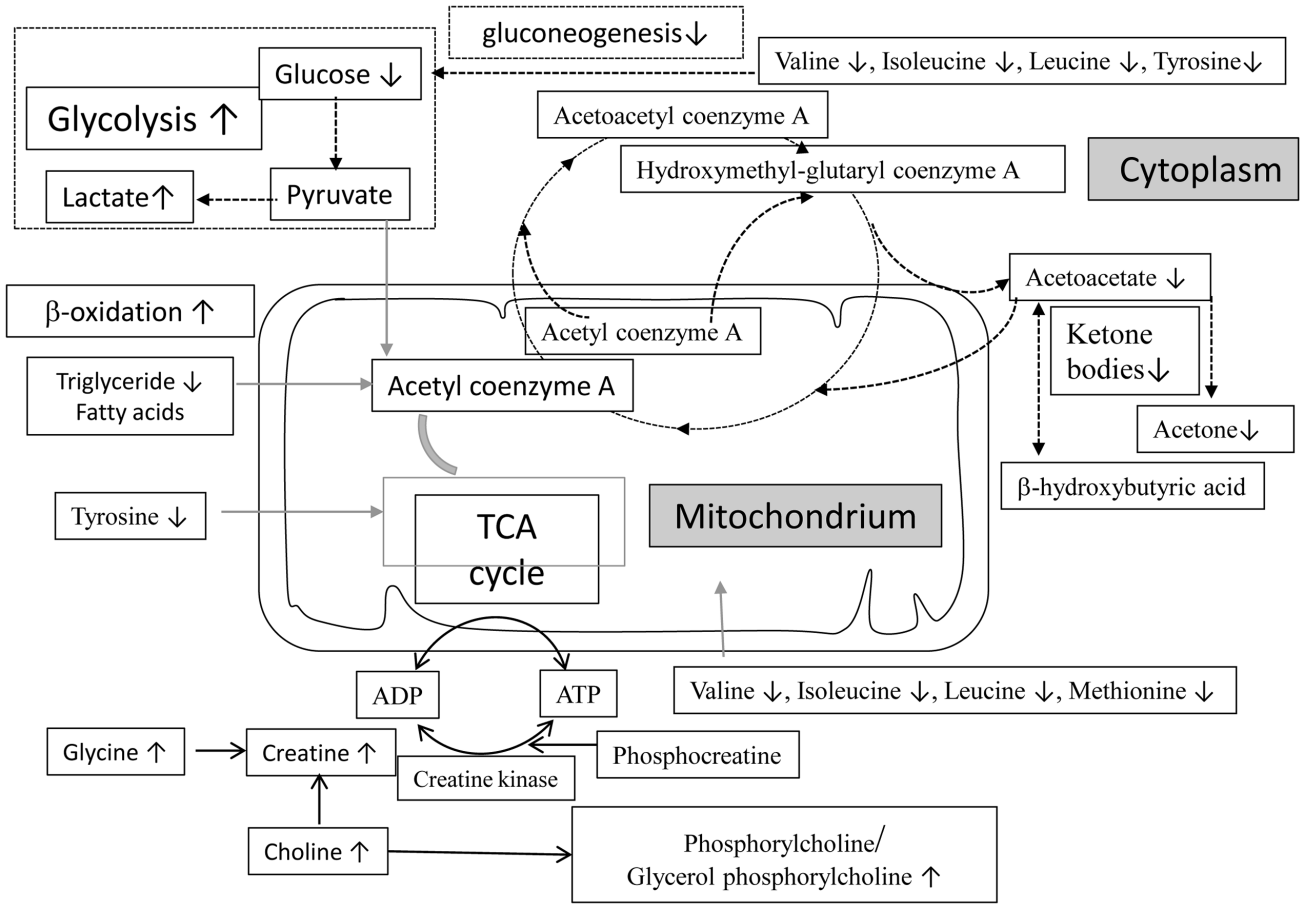
Metabolic changes induced by *M. tb* and *M. bovis* infection prompted a switch in the energy production pathways. Our hypothesis states that the infection induced glycolysis, which led to a decrease in glucose concentration and a lactate accumulation. The decrease in cellular energy levels due to infection triggered lipid degradation. Then, the triacylglycerol and free fatty acids were used as a source of carbon and energy via β-oxidation. Meanwhile, ketone bodies were by-products that contributed to energy production via acetyl coenzyme A. Other energy sources included creatine. The amino acids may contribute to energy production by conversion of the intermediates of the TCA cycle or converted into glucose through gluconeogenesis. ↑ represents markedly increased metabolites, while ↓ representsdecreased metabolites.

In agreement with clinical malnutrition and wasting signs of the infected cattle, the metabolic profiles of the infected cattle in this study showed many indications of shift from energy production, such as glycolysis promotion and utilization of substances other than glucose as an energy source, such as fatty acids, ketone bodies, amino acids, and creatine (Figure 10). Overall, all significantly changes in metabolites observed in the *M. bovis-*infected groups also occurred in the *M. tb*H37Rv-infected group. Furthermore, compared to previous works related to the metabolic profiling of guinea pigs and mice after *M. tb* infection, this study discovered fewer alterations in metabolite profiles [18], [19], [42], which may correspond to pathological severity. In previous studies, experimentally infected guinea pigs successfully developed lung granulomas and mice developed granulomatous inflammation, while infected cattle did not produce a typical TB pathology.

### Carbohydrate metabolism

In all of the groups infected with the three strains (*M. tb*H37Rv, and *M. tb*1458 and *M. bovis*), the glucose levels were decreased and lactate levels were increased compared to the control cattle. A previous report confirmed that in a low glucose environment, *M. tb* could survive in a non-replicating state for longer periods in culture medium, which was in agreement with the persistent infection and chronic process of TB [43]. Lactate is the end product of anaerobic glycolysis and concurrent changes in the levels of glucose and lactate demonstrated glycolysis stimulation were commonly observed in TB models of mice [18] and guinea pigs [19]. Glycolysis stimulation might be attributed to metabolic abnormalities of infected cattle and bacterial metabolic adaptations. First, glycolysis may be enhanced in granulomatous inflammation primarily in macrophages and neutrophils and can be regulated by cytokines, such as IL-1α and β [44]. In the current study, we observed a continuous increase in IL-1β levels and significantly increased neutrophil infiltration, as these immune cells secrete IL-1 in response to infections. These responses may have contributed to the elevated glycolysis in this study. Secondly, in vivo bacterial survival is dependent on the glycolytic pathway. Persistent *M. tb* infection in the host lowers oxygen tension, which, in turn, triggers the glycolytic pathway and increases glucose consumption and concomitant lactate production [45]. In this study, we confirmed the presence of a specific antigen to *M. tb* and *M. bovis* and, therefore, the bacteria induced anaerobic glycolysis and may have also contributed to the overall increase in glycolysis.

### Lipid and fatty acid metabolism

The levels of serum TG and ketone bodies (acetone and acetoacetate) were significantly decreased in the groups infected with either *M. tb*H37Rv or *M. bovis*. These findings indicated that the host cell pathways were diverted to use lipids and fatty acids as carbon substrates to produce energy through β-oxidation in an environment of glucose deficiency and low oxygen. Ketone bodies are by-products of fatty acid β-oxidation and can be transported from the liver to other tissues and reconverted to acetyl-CoA to produce energy via the tricarboxylic acid (TCA) cycle. The utilization of ketone bodies by the *M. tb-* and *M. bovis*-infected cattle in this study was not previously observed in mice or guinea pigs infected with *M. tb* [18], [19].

Creatine, an organic acid that supplies energy to all somatic cells with a balance of adenosine-5′-triphosphate/adenosine-5′-diphosphate (ATP/ADP), was increased in two groups infected with different *M. tb* strains. Under the condition of increased energy demands, the phosphagen system rapidly re-synthesizes ATP from ADP with the use of phosphocreatine through a reversible reaction with the enzyme creatine kinase [46], which was in agreement with the high serum creatine concentrations in the current study, which demonstrated energy redistribution after TB infection.

### Amino acid metabolism

In this study, several amino acids were significantly decreased in all three infected groups, including the BCAAs (isoleucine, leucine, and valine), glycine, methionine, and tyrosine, which indicated that nutrient deprivation obliges the cells to acquire ATP from host-derived proteins and other nutritional sources during glycopenia. Amino acid deprivation can contribute to energy production by conversion of the intermediates of the TCA cycle. For instance, tyrosine can be converted to fumarate and the amino acids valine, isoleucine, leucine, and methionine can be converted to succinate. Therefore, the reduction of these amino acids may represent decreases in the corresponding intermediates of the TCA cycle due to energy deprivation. Actually, the intermediate citrate was significantly decreased in groups infected with the *M. tb* strains, which was consistent with the above concept.

Meanwhile, the BCAAs were typical glucogenic amino acids, which could be converted to glucose via gluconeogenesis during glycopenia. The depletion of BCAAs may also reflect a compensatory mechanism of promoted gluconeogenesis to maintain host energy and homeostasis. Reportedly, without gluconeogenesis, *M. tb* cannot replicate in lung tissue and led to glucose clearance during the chronic phase in a murine model [47]. In the current study, the concentrations of BCAAs were decreased compared to the uninfected control group, which indicated that the bacterium utilized BCAAs to trigger gluconeogenesis for in vivo growth and infection persistence. These results were in accordance with those of a previous study. Similar effects were also found following infections by S*chistosoma* [48], [49] or *Echinostoma* [50]. Regarded as a glucogenic and ketogenic amino acid [51], the decrease in tyrosine content after *M. tb* infection was also an indicator of disrupted energy metabolism.

### Changes in N-acetyl glycoprotein confirmed an inflammatory response

The resonances ranged between 2.04 and 2.08 ppm, which indicated that the spectra originated from *N*-acetyl groups of *N*-acetylated carbohydrate side-chains present in reactive plasma glycoproteins during the acute phase of infection [52]. Compared to the control group, both the *M. bovis*-and *M.tb*1458-infected groups similarly showed a significant increase in *N*-acetyl glycoproteins, which are induced by inflammation and trauma [53], and may be related to the synthesis of vital and characteristic cell wall components of bacteria [54]. This finding was in agreement with actions of other inflammatory markers, such as increased neutrophil infiltration and IL-1β production, in this study.

In addition, elevated choline content was found to be a feature characteristic of neoplastic lesions, such as tuberculomas, fungal granuloma, or tumors via in vivo proton magnetic resonance spectroscopy, probably as a result of accelerated cellular proliferation and metabolism [55]. Although no tuberculomas were observed during the experimental period in this study, the elevated levels of choline and PC in all of the infected cattle indicated that tuberculomas may have developed if the observation period was extended.

In conclusion, by experimental infection, we showed that both *M. tb* and *M. bovis* have similar abilities to infect cattle and subsequently induce inflammation and immune responses, and produce similar abnormal metabolic profiles before the occurrence of typical tubercles or clear granulomatous inflammation in cattle. Therefore, *M. tb* appears to be virulent to cattle.
